# Closing the knowledge‐action gap in conservation with open science

**DOI:** 10.1111/cobi.13835

**Published:** 2021-11-29

**Authors:** Dominique G. Roche, Rose E. O'Dea, Kecia A. Kerr, Trina Rytwinski, Richard Schuster, Vivian M. Nguyen, Nathan Young, Joseph R. Bennett, Steven J. Cooke

**Affiliations:** ^1^ Canadian Centre for Evidence‐Based Conservation, Department of Biology and Institute of Environmental and Interdisciplinary Science Carleton University Ottawa Ontario Canada; ^2^ Institut de Biologie Université de Neuchâtel Neuchâtel Switzerland; ^3^ Evolution & Ecology Research Centre and School of Biological and Environmental Sciences University of New South Wales Sydney New South Wales Australia; ^4^ Canadian Parks and Wilderness Society (CPAWS) ‐ Northern Alberta, Edmonton, Alberta Canada; ^5^ Nature Conservancy of Canada Vancouver British Columbia Canada; ^6^ School of Sociological and Anthropological Studies, Faculty of Social Sciences University of Ottawa Ottawa Ontario Canada; ^7^ Department of Biology Carleton University Ottawa Ontario Canada

**Keywords:** critical appraisal, evidence‐based decision‐making, knowledge mobilization, open access, open code, open data, open education resources, transparency, acceso abierto, código abierto, datos abiertos, movilización del conocimiento, recursos educativos abiertos, toma de decisiones basada en evidencias, transparencia, valuación crítica, 严格评价, 循证决策, 知识动员, 开放存取, 开放代码, 开放数据, 开放教育资源, 透明度

## Abstract

The knowledge‐action gap in conservation science and practice occurs when research outputs do not result in actions to protect or restore biodiversity. Among the diverse and complex reasons for this gap, three barriers are fundamental: knowledge is often unavailable to practitioners and challenging to interpret or difficult to use or both. Problems of availability, interpretability, and useability are solvable with open science practices. We considered the benefits and challenges of three open science practices for use by conservation scientists and practitioners. First, open access publishing makes the scientific literature available to all. Second, open materials (detailed methods, data, code, and software) increase the transparency and use of research findings. Third, open education resources allow conservation scientists and practitioners to acquire the skills needed to use research outputs. The long‐term adoption of open science practices would help researchers and practitioners achieve conservation goals more quickly and efficiently and reduce inequities in information sharing. However, short‐term costs for individual researchers (insufficient institutional incentives to engage in open science and knowledge mobilization) remain a challenge. We caution against a passive approach to sharing that simply involves making information available. We advocate a proactive stance toward transparency, communication, collaboration, and capacity building that involves seeking out and engaging with potential users to maximize the environmental and societal impact of conservation science.

## Abstract (es)

Cierre de la Brecha entre el Conocimiento y la Acción en la Conservación con Ciencia Abierta 21–311

**Resumen:**

La brecha entre el conocimiento y la acción en las ciencias de la conservación y en su práctica ocurre cuando los resultados de las investigaciones no derivan en acciones para proteger o restaurar la biodiversidad. Entre las razones complejas y diversas de esta brecha, existen tres barreras que son fundamentales: con frecuencia el conocimiento no está disponible para los practicantes, es difícil de interpretar o difícil de usar, o ambas. Los problemas con la disponibilidad, interpretabilidad y utilidad son solucionables mediante las prácticas de ciencia abierta. Consideramos los beneficios y los obstáculos de tres prácticas de ciencia abierta para su uso por parte de los científicos y practicantes de la conservación. Primero, las publicaciones de acceso abierto hacen que la literatura científica esté disponible para todos. Segundo, los materiales abiertos (métodos detallados, datos, códigos y software) incrementan la transparencia y el uso de los hallazgos de las investigaciones. Tercero, los recursos educativos abiertos permiten que los científicos y practicantes de la conservación adquieran las habilidades necesarias para utilizar los productos de las investigaciones. La adopción a largo plazo de las prácticas de ciencia abierta ayudaría a los investigadores y a los practicantes a lograr los objetivos de conservación mucho más rápido y de manera eficiente y a reducir las desigualdades que existen en la divulgación de información. Sin embargo, los costos a corto plazo para los investigadores individuales (incentivos institucionales insuficientes para participar en la ciencia abierta y en la movilización del conocimiento) todavía son un reto. Advertimos sobre las estrategias pasivas de divulgación que simplemente hacen que la información esté disponible. Abogamos por una postura proactiva hacia la transparencia, la comunicación, la colaboración y la construcción de las capacidades que incluyen la búsqueda de y la interacción con los usuarios potenciales para maximizar el impacto ambiental y social de las ciencias de la conservación.

## 用开放科学填补保护中的知识‐行动缺口

**【摘要】** 在保护科学和实践中, 当研究成果不能转化为保护或恢复生物多样性的行动时, 就会出现知识‐行动缺口。在造成这种缺口的各种复杂原因中, 有三个基本的障碍:保护实践者往往无法获得知识, 或难以理解, 或难以应用, 又或者两者都有。然而, 可获得性、可解释性和可用性的问题可以通过开放科学实践来解决。我们考虑了三种开放科学实践对保护科学家和实践者的好处和挑战。第一, 开放获取出版使所有人都能获得科学文献。第二, 开放材料(详细的方法、数据、代码和软件)增加了研究结果的透明度和可用性。第三, 开放的教育资源使保护科学家和实践者能够获得使用研究成果所需的技能。长期采用开放科学实践将帮助研究者和实践者更快、更有效地实现保护目标, 并减少信息共享中的不公平现象。然而, 研究者个人的短期成本(参与开放科学和知识动员的制度激励不足)仍然是一项挑战。我们告诫大家不要采取仅提供信息的被动分享方式, 而应对透明度、沟通、合作和能力建设采取积极主动的态度, 包括寻找潜在使用者并与之接触, 从而充分发挥保护科学的环境和社会影响力。**【翻译:胡怡思;审校:聂永刚】**

## INTRODUCTION

**FIGURE 1:**
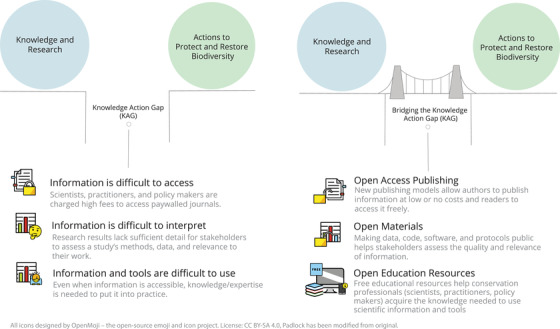
How open science practices can help bridge the knowledge‐action gap in conservation. Artwork by Elise Gagnon, Canadian Parks and Wilderness Society (CPAWS) ‐ Northern Alberta

Conservation science focuses on understanding environmental problems to inform management and policy actions that protect or restore biodiversity (Soulé, 1985). As such, conservation science falls short when research results are not integrated into policy or practice––the so‐called “knowledge‐action gap” (e.g., Knight et al., 2008; Cook et al., 2013; Fabian et al., 2019). Although the largest barrier to putting conservation science into practice remains a lack of political will to implement evidence‐based policies (Young et al., 2016; Bertuol‐Garcia et al., 2018), political and social inertia are not the only obstacles. The inability of policy makers and practitioners to access, interpret, and use knowledge generated by conservation scientists is a key contributor to the knowledge‐action gap (Fuller et al., 2014; Alston, 2019; Walsh et al., 2019; Buxton et al., 2021). It increases the likelihood of practitioners and policy makers basing their decisions on personal experience, anecdotal evidence, or political beliefs rather than scientific evidence (Cook et al., 2010; Fabian et al., 2019). One means for improving how conservation knowledge is accessed, interpreted, and put into practice is to engage in open science (Figure 1).

Open science promotes transparency and reproducibility and aims to strengthen the credibility and usability of research results (McKiernan, 2017; Munafò et al., 2017) and maximize the efficiency and impact of scientific research (and teaching and capacity building) by mobilizing research products beyond the traditional, often opaque, scientific article (O'Dea et al., 2021). Open science practices are spreading across the natural and social sciences through initiatives that increase public access to the scientific literature, encourage comprehensive disclosure of methods, data, and analyses, and promote free access to educational resources. Enhancing transparency should make knowledge more consumable and trusted by a wider variety of audiences and therefore, more useable, narrowing the knowledge‐action gap.

Closing the knowledge‐action gap is insufficient on its own to solve complex policy problems that require political action and negotiation (Sarewitz, 2015). Being able to access and interpret scientific research findings is important for responsible decision‐making, but must be paired with strategies for engaging stakeholders and rights holders who may hold different forms of knowledge (including local, experiential, and Indigenous knowledge) and should be considered in decision‐making (Reid et al., 2021). In this regard, open science is grounded in principles of inclusivity and can foster multidirectional approaches to research (i.e., coproduction).

Support for open science is growing among academic institutions, publishers, funding agencies, and governments (e.g., Moher et al., 2018; Roche et al., 2020; Jarrad et al., 2021). However, motivating researchers to engage actively and meaningfully in open science remains a challenge because of insufficient institutional incentives for researchers to change their behavior (O'Dea et al., 2021). Some academics are reluctant to publicly share their data for fear of receiving insufficient credit or data being misused (Tenopir et al., 2011; Roche et al., 2014). Fortunately, surveys indicate that academics are progressively embracing initiatives to improve transparency (Tenopir et al., 2015; Soeharjono & Roche, 2021). Adopting open science principles is critical for the conservation science community to increase the impact of scientific research on conservation policy and practice and to increase return on conservation investments. We considered three key aspects of open science that will help narrow the knowledge‐action gap––open access publishing, open materials (detailed methods, data, code, and software), and open educational resources––and challenges and opportunities to bolster their uptake.

## OPEN ACCESS PUBLISHING

For conservation knowledge to result in action, it must be available to practitioners and policy makers (Gossa et al., 2015). Currently, institutions pay hefty fees for their employees to access traditional subscription‐only journals. Under this pay‐to‐read model, authors can often publish free of charge, but fiscally challenged institutions and the general public are locked out of academic research unless they pay high fees to access individual articles. Such barriers create disincentives for nonacademic groups and institutions to use new scientific knowledge. Furthermore, by allowing free access online only to the abstract of published articles, paywalls may lead to misinterpretations or misrepresentations of scientific studies.

Demand to democratize scientific knowledge is exemplified by the surge in the use of Sci‐Hub, a shadow library from which tens of millions of scientific articles are illegally downloaded every year (Himmelstein et al., 2018). Sci‐Hub is a workaround to the paywall problem, but it does not solve access issues for lawful institutions and their employees. For example, public servants charged with monitoring and protecting biodiversity often cannot access the conservation literature that was funded by government grants (Larios et al., 2020). Fortunately, the scientific publishing industry is in the midst of a transition to open access publishing, in which reading scientific journal articles and books is free for all (Fuller et al., 2014; Alston, 2019).

Positive outcomes of open access publishing directly align with the fundamental objective of conservation science: to translate research into effective, evidence‐based environmental management and policy (Bolick et al., 2017). When access barriers are broken down, research findings can be taken up by a diversity of stakeholders more readily, including managers, policy makers, citizen scientists, grassroots conservation coalitions, and researchers without institutional subscriptions to scientific journals (Tennant et al., 2016; Piwowar et al., 2018). For this reason, many conservation journals are now fully open access or offer open access options (Alston, 2019). However, making knowledge available does not necessarily make it interpretable (see “Open Materials” and “Open Educational Resources” below).

Because open access publishing facilitates communication among scientists and with the public, a growing number of governments and philanthropic organizations that fund conservation science have mandatory open access policies. For example, the U.S. National Science Foundation (nsf.gov) and the Gordon and Betty Moore Foundation (moore.org) now require that all publications from funded projects be openly accessible. These initiatives make a lot of sense for national funding bodies because sustainable open access models prevent the public from paying for the same research twice: once when funding the research and a second time to access the publication (Table 1).

**TABLE 1:** Open‐access (OA) publishing options available to authors

APC‐based open access Pay to publish, free to read, typesetting done by the publisher or journal Under this model, open‐access journals typically shift the cost of publication from readers to authors via article processing charges or article publication costs (APCs), creating “authorship barriers out of readership barriers” (Bolick et al., 2017). Publishing costs affect where many authors choose to publish their work because APCs can be prohibitively expensive (often ranging from US$1000 to $5000). Under this publishing model, authors pay instead of the readers so that access to scientific articles is unrestricted. Some publishers offer APC discounts or waivers––for example, to authors from lower income countries––yet the pay‐to‐publish open access model is fundamentally inequitable and unsustainable because of funding disparities among research groups, institutions, and regions of the world (Peterson et al., 2019).
Green open access Free to publish, free to read, no typesetting Green open access is achieved by self‐archiving a preprint (e.g., on osf.io and ecoevorxiv.com), making a manuscript free to read even if it is ultimately published in a subscription‐based journal. It is also self‐archiving of the peer‐reviewed, revised, accepted version of a manuscript before editing and typesetting in a public repository (i.e., a postprint). The SHERPA RoMEO database provides information on which journals authorize self‐archiving and under what circumstances (http://sherpa.ac.uk/romeo). In opting for green OA, conservation scientists ensure that their results can reach end‐users at no direct cost to their lab or research program.
Diamond or platinum open access Free to publish, free to read, typesetting done by the publisher or journal Diamond (a.k.a. platinum) open access journals rely on funding sources, such as funder or society subsidies, consortium funding from libraries, lifetime author subscriptions (e.g., PeerJ), or some creative combination of these options, to cover publishing costs (Bolick et al. 2017; Willinsky & Rusk, 2019). Diamond OA embraces a social mission that is compatible with not‐for‐profit publishers. A notable example of a successful diamond open access initiative is Redalyc (https://www.redalyc.org), which has existed since 2003 and supports >1400 journals in Latin America. In total, an estimated 29,000 diamond open access journals exist (Science Europe, 2021).
Overlay journal Free to publish or low‐cost pay to publish, free to read, typesetting done by authors or journal Overlay journals rely on free‐to‐use preprint servers (e.g., Arχiv and bioRχiv). They have a website, an editorial board, and rely on volunteer reviewers. Authors upload their manuscript to a preprint server and submit the link to an overlay journal of their choice (typically discipline specific). The journal sends the preprint for conventional peer review. When a paper is accepted, the overlay journal website publishes a link to the final version of the paper on the preprint server at no cost to authors or readers. To keep production costs down, overlay journals ask authors to do their own typesetting or use a free or low‐cost journal management platform to do so. For example, *Discrete Analysis*, an overlay journal in mathematics, uses Scholastica for typesetting (https://scholasticahq.com). Production costs are US$10 per article and are covered by a small grant from the University of Cambridge (Ball, 2015). For a list of existing overlay journals, see Mounce (2021).

Beyond societal benefits, open access benefits authors by increasing the reach and impact of their work: open access studies tend to be cited more often in the scientific literature, communicated more frequently in traditional and social media, and referenced more often in policy documents (e.g., Eysenbach, 2006; Gargouri et al., 2010; Tai & Robinson, 2018). Despite these advantages and considerable growth in open access publishing (Piwowar et al., 2018), broader adoption is hindered by perceptions of lower status and the financial cost to authors.

The belief among authors and academic institutions that open access journals are of lower status than their subscription‐only counterparts stymies the adoption of open access. To cover their publication costs––and in some cases generate profits––open access journals typically charge authors an article processing charge (APC) (Table 1). Perceptions that these pay‐to‐publish journals have lower standards have been exacerbated by innovations, such as rapid review turnaround times and online‐only publication (Ware & Mabe, 2015), and predatory open access journals (outlets that publish articles for a fee with virtually no reviewer or editorial oversight) (Grudniewicz et al., 2019; Siler, 2020). Although predatory journals represent a small fraction of regularly cited open access outlets (Olijhoek & Tennant, 2018) and tools exist to identify trustworthy open access journals (DOAJ, 2019; Grudniewicz et al., 2019), predatory journals cast a shadow over open access publishing. For example, in the United States and Canada, *open access* was mentioned in only 5% of review, promotion, and tenure documents from across 129 universities in 2017, and most of these mentions discouraged authors from publishing in such outlets (Alperin et al., 2019). Still, perceptions of open access journals continue to improve as publishing features introduced by online‐only journals become normalized and as established publishers offer high‐status open access journals (e.g., *Nature Communications*, *Science Advances*, and *PLOS Biology*).

Once open access journals overcome negative perceptions, cost is still a problem (Table 1). An alternative to APCs is the green OA option of self‐archiving a pre‐ or postprint in a public repository (Table 1). Other models are also emerging that aim to radically change open access publishing and make research free to read and free to publish (Table 1). With the increasing number of open access journals and emergence of new publication styles, the goal of making research available for all is within reach. The next step in closing conservation's knowledge‐action gap is to make published research interpretable.

## OPEN MATERIALS

To close the knowledge‐action gap, conservation science should be reported in sufficient detail for scientists and practitioners to assess the quality of the research and its relevance to their goals (Roche et al., 2019; Bocking, 2020). Evaluating research for its reliability and relevance (i.e., critical appraisal) helps one determine whether a study has internal validity (e.g., representative sampling, appropriate methods of measurement, and robust statistical analyses) (Josefsson et al., 2020). Assessments of study relevance (i.e., external validity) require descriptions of methods that are sufficient for one to determine whether study results are likely generalizable and pertinent to a particular question (Cooke et al., 2017a). For example, one should be able to determine how similar the population or environmental conditions are to those in the system of interest. Critical appraisal is an essential component of evidence‐based decision‐making, but it is technically and practically challenging, and methods are still being refined (see Collaboration for Environmental Evidence [www.environmentalevidence.org/cee‐critical‐appraisal‐tool]). Incomplete and opaque reporting is a fundamental barrier to critical appraisal (Josefsson et al., 2020) that contributes to the knowledge‐action gap.

Critical appraisal relies on researchers providing open detailed methods, data, and code, that is, the materials necessary to reproduce and/or replicate a study. Open methods means the methods are comprehensively and transparently reported, such that readers know what was done and can replicate or extend the methods (Munafò et al., 2017). Open data allow readers to verify whether conclusions are backed by the data, support long‐term monitoring and comparative studies, and facilitate evidence synthesis (Costello & Wieczorek, 2014; Haddaway, 2015; Culina et al., 2018b). Open data also allow researchers to consider new questions, often at a broader scale, to build knowledge and understanding (Tenopir et al., 2011; Poisot et al., 2013) (Appendix S1). Ideally, open data are provided in an unprocessed and user‐friendly format alongside informative metadata (complete descriptions, including the meaning of variable names and units) and contain all examined variables because potential users may be interested in data that were not the primary focus of a study (White et al., 2013; Costello & Wieczorek, 2014). Open code allows readers to check whether the results reported can be reproduced with the software used by the authors. Open code includes the code or script used to process raw data, conduct statistical analyses, and execute simulation or computational‐based models (Barnes, 2010; Stodden et al., 2016; Culina et al., 2020).

Open materials also help generate conservation knowledge more quickly, efficiently, and equitably (Buxton et al., 2021). Openly sharing information allows conservation scientists and practitioners to build on each other's work, learn about new tools and techniques more quickly, and avoid repeating others’ mistakes (Molloy, 2011; Lowndes et al., 2017). Additionally, open materials help improve citizen science initiatives, which contribute invaluable data to conservation science (e.g., Sullivan et al., 2017; Robinson et al., 2020). Open data also ensure maximum benefits from the costs of data collection (Costello et al., 2013; Hampton et al., 2013; Turner et al., 2015). For example, open data make it easier to find information on a target species or ecosystem (Culina et al., 2018a); they facilitate evidence synthesis (especially meta‐analysis), particularly for species that are less‐well studied (Culina et al., 2018b); and they allow better‐designed and hence more informative studies (e.g., by facilitating power analyses to avoid errors of statistical significance and effect sign or magnitude) (Gelman & Carlin, 2014) (Appendix S1). Overall, open materials help convert conservation knowledge into action by reducing inequalities among research groups and nations and preventing knowledge from being lost in institutional ivory towers (Stodden, 2010; Carillo & Papagni, 2014; Rey, 2014). In addition to open materials, other open resources focus on addressing the challenge of synthesizing complex information to inform environmental decision‐making (Appendix S2).

Open materials is a seemingly simple concept that is nonetheless challenging for researchers to implement (Gewin, 2016; Perkel, 2018) and difficult for practitioners to engage with. Although researchers may try their best to provide open methods, data, and code, their records may not provide adequate details (Haddaway & Verhoeven, 2015), files might not be stored in a location or format that will be usable in the long‐term (Poisot et al., 2019), and analysis scripts may fail to run after software updates (Perkel, 2019). Ensuring long‐term benefits of open data and code requires that researchers be taught how to adhere to FAIR principles: data and code are findable (readily found with a keyword search), accessible (accessible by the public), interoperable (data can be imported and understood; code can be run on another computer with nonproprietary software), and usable (information can be understood and results reproduced) (www.go‐fair.org; Wilkinson et al., 2016). For long‐term benefits, funders and end‐users of conservation science should require funded projects to produce FAIR outputs.

The process of preparing and publicly sharing materials typically occurs only after a study is completed, but it is easier to produce these materials if the entire research project is conducted transparently from the onset. Alston and Rick (2020), Buxton et al. (2021), and Kathawalla et al. (2021) provide practical guidance on how to engage in these practices. Preregistrations and registered reports encourage “process transparency” (rather than post hoc transparency) because they provide records of research plans (Parker et al., 2019). In a registered report, the plan is submitted to a journal for peer review and can be provisionally accepted prior to knowing the results (Nosek & Lakens, 2014; Parker et al., 2019). For conservation practitioners, process transparency provides the opportunity to suggest changes to a plan that could improve the study's value for conservation (i.e., a multidirectional approach to research) and to request styles of research output tailored to their needs (e.g., open software tools), in addition to interpretable and useable scientific articles (Stodden, 2010).

Beyond open materials, conservation scientists can actively engage practitioners with open software (Appendix S3) and communication tools. The time and training required to work with open data and code can be prohibitive, but website and desktop applications (e.g., R Shiny and Code Ocean) allow practitioners to reap many benefits from this shared information (Whitehead & Booker, 2019). Because open software tools are more engaging and user friendly than static files, they can facilitate communication between conservation researchers, practitioners, stakeholders, and advocacy groups, thereby helping to close the knowledge‐action gap. For example, in Canada, the Pacific Salmon Foundation harvests data from various sources to assess the status of unique salmon populations and risks to their habitat from human and environmental threats. The Foundation's Pacific Salmon Explorer tool (http://salmonexplorer.ca), which allows visualization of these data, has played a key role in shaping Canada's Department of Fisheries and Oceans’ Wild Salmon Policy Implementation Plan (www.pac.dfo‐mpo.gc.ca/consultation/wsp‐pss/index‐eng.html). Infographics, other engaging and educational visuals, and research summaries written for the general public are also outputs that researchers can use to engage with a wider audience. Beyond the traditional academic paper, conservation research could reach a much wider audience if scientists were incentivized to produce practical outputs and share them with the general public and practitioners. These outputs can receive permanent identifiers (e.g., a DOI) that allow them to be cited and the authors credited (Nosek et al., 2015 [https://www.cos.io/initiatives/top‐guidelines]).

Aside from the practical challenges of producing open and useable materials in conservation science, there are issues of ethics, confidentiality, and ownership (e.g., Nguyen et al., 2017). Ethically, some information needs to be withheld if it could be exploited for nefarious purposes, such as the illegal wildlife trade (Cooke et al., 2017b). Confidentiality issues arise when conservation science intersects with human communities and the identity of human subjects needs to be anonymized (Pérignon et al., 2019). In the case of Indigenous knowledges, at a minimum researchers must comply with the OCAP (https://fnigc.ca) and CARE (https://www.gida‐global.org/care) principles. Every Indigenous community and government is different, so researchers must consult with communities to determine how Indigenous knowledge or data collected from their territories are archived and shared (Wong et al., 2020). Moreover, conflicts over data sharing could arise when conservation scientists collaborate with private entities. For these reasons and others, it may not always be possible or beneficial for all methods or data from conservation research to be open. In these cases, access control or partially open materials is the next best option (Lowe et al., 2017; Lennox et al., 2020).

## OPEN EDUCATION RESOURCES

Opening access to conservation research outputs, whether scientific articles or research materials, has limited benefits for protecting biodiversity if practitioners are unable to use these resources or the results are not relevant. Adequately interpreting and using research outputs requires diverse skill sets, including familiarity with experimental design, data acquisition methods, and literacy in statistical analysis, computer programing, software use, and science communication. Much like scientists who receive little training in knowledge mobilization and stakeholder engagement, conservation practitioners may lack the skills to implement the findings and use the tools produced through conservation research. Fortunately, information technology provides conservation professionals (scientists, practitioners, and policy makers) the opportunity to acquire relevant skills freely and flexibly through open education resources (OERs).

The term *OER* refers to digital materials released under an open license, which allows them to be freely accessed, retained, remixed, revised, reused, and redistributed (the five Rs) for teaching, learning, and research (UNESCO, 2019). Examples include presentation slides, textbooks, audio and video lectures, course syllabi, protocols, data sets, and scripts on a wide range of topics, such as data, computer, and environmental science. For instance, conservation professionals wanting to learn about artificial intelligence (AI) can access open textbooks and online classes offered on several OER platforms (Table 1). Open education resources can help developers of conservation tools train end users and save time through reuse or remixing of existing materials.

Technology is often celebrated for bringing new methods of measurement and data analysis to conservation science and practice (Berger‐Tal & Lahoz‐Monfort, 2018). Common examples are biologging, image recognition (computer vision and AI), remote sensing, aerial monitoring, and platforms enabling community science (e.g., iNaturalist and eBird). However, conservation professionals often overlook the potential for new technologies to democratize education and training that would allow broader and more efficient implementation of conservation tools and solutions.

Few OERs exist that specifically focus on conservation (but see Downey et al., 2021 and https://ncep.amnh.org), yet existing OERs can help conservation practitioners interpret and implement research outputs in conservation science (e.g., OERs on computer science, data science, economics, engineering, and communication). Finding these resources through centralized databases and search engines is straightforward (Table 1). For example, the Open Courseware Consortium (oeconsortium.org), an international network of open education organizations, builds capacity for finding, reusing, creating, and sharing OERs. The consortium uses the MERLOT system (merlot.org), which allows users to search a curated database of OERs from across >4000 member institutions. In addition to these courses, massive open online courses (MOOCs) (mooc.org) allow one to earn credits toward academic degrees––MOOCs are free but some fees apply for earning credits. The Open Science MOOC (Table 2) is notable for allowing conservation students and researchers gain knowledge in transparent and reproducible research practices.

**TABLE 2:** Platforms for accessing open education resources (OERs)

Platform	URL
American Museum of Natural History ‐ Network of Conservation Educators and Practitioners	https://ncep.amnh.org
British Columbia Open Education	https://open.bccampus.ca
CC Open Education Platform	https://network.creativecommons.org/cc‐open‐education‐platform
Chromebook Data Science	https://jhudatascience.org/chromebookdatascience
Coursera	https://www.coursera.org
Coursera	https://www.coursera.org
eCampusOntario	https://openlibrary.ecampusontario.ca
edX	https://www.edx.org/
Evidence in Conservation Teaching	http://bit.ly/Evidence‐in‐Conservation‐Teaching
Massive Open Online Courses	https://www.mooc.org
MIT OpenCourseWare	http://ocw.mit.edu
OER Africa	http://www.oerafrica.org
OER Commons	https://www.oercommons.org
OER University	https://oeru.org
Open Oregon Educational Resources	https://openoregon.org
Open Science MOOC	https://opensciencemooc.eu
Open Textbook Library	https://open.umn.edu/opentextbooks
Open Yale Courses	https://oyc.yale.edu/
OpenLearn initiative	http://openlearn.open.ac.uk
Openlearn of OUUK	https://www.open.edu/openlearn
OpenStax (Rice University)	https://openstax.org
Stanford on iTunes	https://cardinalatwork.stanford.edu/benefits‐rewards/sweeteners/stanford‐itunes‐u
WISElearn Resources	https://wlresources.dpi.wi.gov

The OERs will be most effective at closing the knowledge‐action gap in conservation science when they are broadly accessible, reusable, and require a range of time commitments. As with the primary scientific literature in conservation (Amano et al., 2016), OERs are typically published in English, reducing their accessibility to conservation professionals in non‐English‐speaking countries (Krelja Kurelovic, 2016). Ironically, many of these countries contain the world's richest biodiversity hotspots (Myers et al., 2000). Some institutions offer OERs in languages other than English––for example, several OERs on Massachusetts Institute of Technology's (MIT) OpenCourseWare are translated in traditional Chinese, Korean, or Turkish, but accessibility in other languages remains limited. In terms of reusability, OERs should have as few legal permission barriers as possible so that users can readily engage in the five Rs provided the original authors are credited. There has been much progress in this area with the uptake of open license systems (e.g., the Creative Commons, GNU Free Documentation, and MIT licenses), not just for OERs, but also for open materials (https://choosealicense.com). For conservation practitioners who work in small nonprofit organizations, time availability can be a major barrier to accessing conservation science. Encouraging the development of OERs that require a range of time investments, from short summary videos to multiday courses, can increase the use of these resources.

The OERs are attractive to users who can study on their own time and at no cost, but incentives are lacking for those who create them, particularly academics. With little recognition by funders or universities, faculty members already struggling for time might lack the motivation to develop and maintain OER content (Yuan et al., 2008). Ultimately, evidence that OERs can reduce teaching demands on academics and increase the international standing of universities will be key in promoting their broader adoption. In conservation science and practice, an encouraging and important step in this direction is the recent launch of the Evidence in Conservation Teaching Initiative (http://bit.ly/Evidence‐in‐Conservation‐Teaching). This joint effort by over 100 educators across 23 countries provides OER courses focused on the principles and practice of evidence‐based conservation (Downey et al., 2021). In‐depth materials on conservation‐related topics, such as meta‐analysis, how to design management interventions as experiments, and how to use the Conservation Evidence database are also provided.

Finally, direct outreach by researchers to practitioners who are likely to use the results, preferably with engaging summaries or visuals that can be shared, increases awareness of the research among time‐strapped practitioners. These relationships also increase dialogue among researchers and practitioners that will ultimately increase the relevance of research and make it more likely to be incorporated into policy.

## CONCLUSION

Open science can help bridge the knowledge‐action gap in conservation by making scientific information readily available, interpretable, and useable. Importantly, however, open science remains a passive approach to information sharing and therefore, must be coupled with active communication, engagement, and outreach. For example, openly sharing data from conservation research facilitates data scrutiny and reuse, but it might fail to engage relevant end users who could be unaware of the data or lack the capacity to use them. Post hoc transparency (sharing as an afterthought) does not bring about the same credibility as process transparency (planning for transparency from the onset of a project), which is best achieved through early collaboration and knowledge coproduction (Buxton et al., 2021). Conservation practitioners and policy makers use knowledge they trust, and trust is built through confidence and familiarity with both research and researchers (Young et al., 2016). When used in concert, transparency and engagement help build the interpersonal relationships that encourage a multidirectional dialogue between researchers and end users of conservation science to close the knowledge‐action gap by combining forces to solve important conservation problems. Open science is an important step toward increasing that transparency and trust and thus promoting the credibility and legitimacy of one's research and expertise.

Most conservation science is carried out by researchers from high‐income countries, yet the world's greatest conservation needs tend to occur in countries with comparatively fewer resources (Hickisch et al., 2019). Open science can help bridge the knowledge‐action gap through equitable information sharing among the world's regions and thus facilitate conservation action where it is most needed. Adopting open science practices requires effort but it can be done in a stepwise fashion, as opposed to an all or nothing approach (Kathawalla et al., 2021). Incremental steps, such as publishing open access, sharing research materials, visual research summaries, and teaching materials, can go a long way toward improving how conservation science translates into practice (see Tai & Robinson, 2018). Open education resources hold enormous potential to boost capacity building and facilitate knowledge mobilization to enable effective conservation action. Finally, assisting conservation scientists in making their research outputs and teaching materials openly accessible requires adequate institutional incentives (Allen & Mehler, 2019; O'Dea et al., 2021). Current reward structures in academia focus overwhelmingly on journal prestige and high publication counts, with key performance indicators failing to capture the long‐term goals of conservation science (Buxton et al., 2021). Given the enormity of the problem of biodiversity loss, a minimum expectation of those trying to address this problem should be research that is accessible, interpretable, and useable. To ensure evidence‐based decision‐making in protecting biodiversity, closing the knowledge‐action gap requires opening science.

## Supporting information

Supporting Information
Additional information is available online in the Supporting Information section at the end of the online article. The authors are solely responsible for the content and functionality of these materials. Queries (other than absence of the material) should be directed to the corresponding author.
Appendix S1. Example of open data for conservation science and practice: the TEAM Network
Appendix S2. Open resources to help with evidence synthesis in conservation science and practice
Appendix S3. Example of open software for conservation science and practice: Marxan Connect

## References

[cobi13835-bib-0001] Allen, C. , & Mehler, D. M. A. (2019). Open science challenges, benefits and tips in early career and beyond. PLoS Biology, 17, e3000246. 31042704 10.1371/journal.pbio.3000246 PMC6513108

[cobi13835-bib-0002] Alperin, J. P. , Muñoz Nieves, C. , Schimanski, L. A. , Fischman, G. E. , Niles, M. T. , & Mckiernan, E. C. (2019). Meta‐research: How significant are the public dimensions of faculty work in review, promotion and tenure documents? eLife, 8, e42254. 30747708 10.7554/eLife.42254 PMC6391063

[cobi13835-bib-0003] Alston, J. M. (2019). Open access principles and practices benefit conservation. Conservation Letters, 12, e12672.

[cobi13835-bib-0004] Alston, J. M. , & Rick, J. A. (2020). A beginner's guide to conducting reproducible research. Bulletin of the Ecological Society of America, 102(2), e01801.

[cobi13835-bib-0005] Amano, T. , González‐Varo, J. P. , & Sutherland, W. J. (2016). Languages are still a major barrier to global science. PLoS Biology, 14, e2000933. 28033326 10.1371/journal.pbio.2000933 PMC5199034

[cobi13835-bib-0006] Ball, P. (2015). Leading mathematician launches arXiv'overlay'journal. Nature News, 526, 146.

[cobi13835-bib-0007] Barnes, N. (2010). Publish your computer code: It is good enough. Nature, 467, 753–753. 20944687 10.1038/467753a

[cobi13835-bib-0008] Berger‐Tal, O. , & Lahoz‐Monfort, J. J. (2018). Conservation technology: The next generation. Conservation Letters, 11, e12458.

[cobi13835-bib-0009] Bertuol‐Garcia, D. , Morsello, C. N. , El‐Hani, C. , & Pardini, R. (2018). A conceptual framework for understanding the perspectives on the causes of the science–practice gap in ecology and conservation. Biological Reviews, 93, 1032–1055. 29160024 10.1111/brv.12385

[cobi13835-bib-0010] Bocking, S. (2020). Science and conservation: A history of natural and political landscapes. Environmental Science & Policy, 113, 1–6.

[cobi13835-bib-0011] Bolick, J. , Emmett, A. , Greenberg, M. L. , Rosenblum, B. , & Peterson, A. T. (2017). How open access is crucial to the future of science. Journal of Wildlife Management, 81, 564–566.

[cobi13835-bib-0012] Buxton, R. T. , Nyboer, E. A. , Pigeon, K. E. , Raby, G. D. , Rytwinski, T. , Gallagher, A. J. , Schuster, R. , Lin, H. ‐. Y. , Fahrig, L. , Bennett, J. R. , Cooke, S. J. , & Roche, D. G. (2021). Avoiding wasted research resources in conservation science. Conservation Science and Practice, 3, e329.

[cobi13835-bib-0013] Carillo, M. R. , & Papagni, E. (2014). “Little science” and “big science”: The institution of “open science” as a cause of scientific and economic inequalities among countries. Economic Modelling, 43, 42–56.

[cobi13835-bib-0014] Cook, C. N. , Hockings, M. , & Carter, R. W. (B). (2010). Conservation in the dark? The information used to support management decisions. Frontiers in Ecology and the Environment, 8, 181–186.

[cobi13835-bib-0015] Cook, C. N. , Mascia, M. B. , Schwartz, M. W. , Possingham, H. P. , & Fuller, R. A. (2013). Achieving conservation science that bridges the knowledge–action boundary. Conservation Biology, 27, 669–678. 23574343 10.1111/cobi.12050 PMC3761186

[cobi13835-bib-0016] Cooke, S. J. , Birnie‐Gauvin, K. , Lennox, R. J. , Taylor, J. J. , Rytwinski, T. , Rummer, J. L. , Franklin, C. E. , Bennett, J. R. , & Haddaway, N. R. (2017a). How experimental biology and ecology can support evidence‐based decision‐making in conservation: Avoiding pitfalls and enabling application. Conservation Physiology, 5, cox043. 28835842 10.1093/conphys/cox043 PMC5550808

[cobi13835-bib-0017] Cooke, S. J. , Nguyen, V. M. , Kessel, S. T. , Hussey, N. E. , Young, N. , & Ford, A. T. (2017b). Troubling issues at the frontier of animal tracking for conservation and management. Conservation Biology, 31, 1205–1207. 28079282 10.1111/cobi.12895

[cobi13835-bib-0018] Costello, M. J. , Michener, W. K. , Gahegan, M. , Zhang, Z.‐Q. , & Bourne, P. E. (2013). Biodiversity data should be published, cited, and peer reviewed. Trends in Ecology & Evolution, 28, 454–461. 23756105 10.1016/j.tree.2013.05.002

[cobi13835-bib-0019] Costello, M. J. , & Wieczorek, J. (2014). Best practice for biodiversity data management and publication. Biological Conservation, 173, 68–73.

[cobi13835-bib-0020] Culina, A. , Baglioni, M. , Crowther, T. W. , Visser, M. E. , Woutersen‐Windhouwer, S. , & Manghi, P. (2018a). Navigating the unfolding open data landscape in ecology and evolution. Nature Ecology & Evolution, 2, 420–426. 29453350 10.1038/s41559-017-0458-2

[cobi13835-bib-0021] Culina, A. , Crowther, T. W. , Ramakers, J. J. , Gienapp, P. , & Visser, M. E. (2018b). How to do meta‐analysis of open datasets. Nature Ecology & Evolution, 2, 1053–1056. 29915339 10.1038/s41559-018-0579-2

[cobi13835-bib-0022] Culina, A. , Van Den Berg, I. , Evans, S. , & Sánchez‐Tójar, A. (2020). Low availability of code in ecology: A call for urgent action. PLoS Biology, 18, e3000763. 32722681 10.1371/journal.pbio.3000763 PMC7386629

[cobi13835-bib-0023] DOAJ . (2019). Directory of Open Access Journals. www.doaj.org

[cobi13835-bib-0024] Downey, H. , Amano, T. , Cadotte, M. , Cook, C. N. , Cooke, S. J. , Haddaway, N. R. , Jones, J. P. G. , Littlewood, N. , Walsh, J. C. , Abrahams, M. I. , Adum, G. , Akasaka, M. , Alves, J. A. , Antwis, R. E. , Arellano, E. C. , Axmacher, J. , Barclay, H. , Batty, L. , Benítez‐López, A. , & Bennett, J. R. (2021). Training future generations to deliver evidence‐based conservation and ecosystem management. Ecological Solutions and Evidence, 2, e12032.

[cobi13835-bib-0025] Eysenbach, G. (2006). Citation advantage of open access articles. PLoS Biology, 4, e157. 16683865 10.1371/journal.pbio.0040157 PMC1459247

[cobi13835-bib-0026] Fabian, Y. , Bollmann, K. , Brang, P. , Heiri, C. , Olschewski, R. , Rigling, A. , Stofer, S. , & Holderegger, R. (2019). How to close the science‐practice gap in nature conservation? Information sources used by practitioners. Biological Conservation, 235, 93–101.

[cobi13835-bib-0027] Fuller, R. A. , Lee, J. R. , & Watson, J. E. M. (2014). Achieving open access to conservation science. Conservation Biology, 28, 1550–1557. 25158824 10.1111/cobi.12346 PMC4241051

[cobi13835-bib-0028] Gargouri, Y. , Hajjem, C. , Larivière, V. , Gingras, Y. , Carr, L. , Brody, T. , & Harnad, S. (2010). Self‐selected or mandated, open access increases citation impact for higher quality research. PLoS One, 5, e13636. 20976155 10.1371/journal.pone.0013636 PMC2956678

[cobi13835-bib-0029] Gelman, A. , & Carlin, J. (2014). Beyond power calculations: Assessing type S (sign) and type M (magnitude) errors. Perspectives on Psychological Science, 9, 641–651. 26186114 10.1177/1745691614551642

[cobi13835-bib-0030] Gewin, V. (2016). Data sharing: An open mind on open data. Nature, 529, 117–119. 26744755 10.1038/nj7584-117a

[cobi13835-bib-0031] Gossa, C. , Fisher, M. , & Milner‐Gulland, E. J. (2015). The research–implementation gap: How practitioners and researchers from developing countries perceive the role of peer‐reviewed literature in conservation science. Oryx, 49, 80–87.

[cobi13835-bib-0032] Grudniewicz, A. , Moher, D. , Cobey, K. D. , Bryson, G. L. , Cukier, S. , Allen, K. , Ardern, C. , Balcom, L. , Barros, T. , Berger, M. , Ciro, J. B. , Cugusi, L. , Donaldson, M. R. , Egger, M. , Graham, I. D. , Hodgkinson, M. , Khan, K. M. , Mabizela, M. , Manca, A. , & Milzow, K. (2019). Predatory journals: No definition, no defence. Nature, 576, 210–212. 31827288 10.1038/d41586-019-03759-y

[cobi13835-bib-0033] Haddaway, N. R. (2015). A call for better reporting of conservation research data for use in meta‐analyses. Conservation Biology, 29, 1242–1245. 25588313 10.1111/cobi.12449

[cobi13835-bib-0034] Haddaway, N. R. , & Verhoeven, J. T. A. (2015). Poor methodological detail precludes experimental repeatability and hampers synthesis in ecology. Ecology and Evolution, 5, 4451–4454. 26664691 10.1002/ece3.1722 PMC4667817

[cobi13835-bib-0035] Hampton, S. E. , Strasser, C. A. , Tewksbury, J. J. , Gram, W. K. , Budden, A. E. , Batcheller, A. L. , Duke, C. S. , & Porter, J. H. (2013). Big data and the future of ecology. Frontiers in Ecology and the Environment, 11, 156–162.

[cobi13835-bib-0036] Hickisch, R. , Hodgetts, T. , Johnson, P. J. , Sillero‐Zubiri, C. , Tockner, K. , & Macdonald, D. W. (2019). Effects of publication bias on conservation planning. Conservation Biology, 33, 1151–1163. 30957293 10.1111/cobi.13326

[cobi13835-bib-0037] Himmelstein, D. S. , Romero, A. R. , Levernier, J. G. , Munro, T. A. , Mclaughlin, S. R. , Greshake Tzovaras, B. , & Greene, C. S. (2018). Sci‐Hub provides access to nearly all scholarly literature. eLife, 7, e32822. 29424689 10.7554/eLife.32822 PMC5832410

[cobi13835-bib-0038] Jarrad, F. , Main, E. , & Burgman, M. (2021). Increasing transparency through open science badges. Conservation Biology, 35, 764–765. 10.1111/cobi.13735 33830537

[cobi13835-bib-0039] Josefsson, J. , Hiron, M. , Arlt, D. , Auffret, A. G. , Berg, Å. , Chevalier, M. , Glimskär, A. , Hartman, G. , Kačergytė, I. , Klein, J. , Knape, J. , Laugen, A. T. , Low, M. , Paquet, M. , Pasanen‐Mortensen, M. , Rosin, Z. M. , Rubene, D. , Żmihorski, M. , & Pärt, T. (2020). Improving scientific rigour in conservation evaluations and a plea deal for transparency on potential biases. Conservation Letters, 13, e12726.

[cobi13835-bib-0040] Kathawalla, U. ‐. K. , Silverstein, P. , & Syed, M. (2021). Easing into open science: A guide for graduate students and their advisors. Collabra: Psychology, 7(1), 18684.

[cobi13835-bib-0041] Knight, A. T. , Cowling, R. M. , Rouget, M. , Balmford, A. , Lombard, A. T. , & Campbell, B. M. (2008). Knowing but not doing: Selecting priority conservation areas and the research–implementation gap. Conservation Biology, 22, 610–617. 18477033 10.1111/j.1523-1739.2008.00914.x

[cobi13835-bib-0042] Kurelovic, E. K. (2016). Advantages and limitations of usage of open educational resources in small countries. International Journal of Research in Education and Science, 2, 136–142.

[cobi13835-bib-0202] Larios, D. , Brooks, T. M. , Macfarlane, N. B. W. , & Roy, S. (2020). Access to scientific literature by the conservation community. PeerJ, 8, e9404 10.7717/peerj.9404 32714657 PMC7354838

[cobi13835-bib-0043] Lennox, R. J. , Harcourt, R. , Bennett, J. R. , Davies, A. , Ford, A. T. , Frey, R. M. , Hayward, M. W. , Hussey, N. E. , Iverson, S. J. , Kays, R. , Kessel, S. T. , Mcmahon, C. , Muelbert, M. , Murray, T. S. , Nguyen, V. M. , Pye, J. D. , Roche, D. G. , Whoriskey, F. G. , Young, N. , & Cooke, S. J. (2020). A novel framework to protect animal data in a world of ecosurveillance. Bioscience, 70, 468–476.

[cobi13835-bib-0044] Lowe, A. J. , Smyth, A. K. , Atkins, K. , Avery, R. , Belbin, L. , Brown, N. , Budden, A. E. , Gioia, P. , Guru, S. , Hardie, M. , Hirsch, T. , Hobern, D. , La Salle, J. , Loarie, S. R. , Miles, M. , Milne, D. , Nicholls, M. , Rossetto, M. , Smits, J. , & Sparrow, B. (2017). Publish openly but responsibly. Science, 357, 141. 28706032 10.1126/science.aao0054

[cobi13835-bib-0045] Lowndes, J. S. S. , Best, B. D. , Scarborough, C. , Afflerbach, J. C. , Frazier, M. R. , O'Hara, C. C. , Jiang, N. , & Halpern, B. S. (2017). Our path to better science in less time using open data science tools. Nature Ecology & Evolution, 1, 0160. 10.1038/s41559-017-0160 28812630

[cobi13835-bib-0046] Mckiernan, E. C. (2017). Imagining the “open” university: Sharing scholarship to improve research and education. PLoS Biology, 15, e1002614. 29065148 10.1371/journal.pbio.1002614 PMC5655613

[cobi13835-bib-0047] Moher, D. , Naudet, F. , Cristea, I. A. , Miedema, F. , Ioannidis, J. P. A. , & Goodman, S. N. (2018). Assessing scientists for hiring, promotion, and tenure. PLoS Biology, 16, e2004089. 29596415 10.1371/journal.pbio.2004089 PMC5892914

[cobi13835-bib-0048] Molloy, J. C. (2011). The open knowledge foundation: Open data means better science. PLoS Biology, 9, e1001195. 22162946 10.1371/journal.pbio.1001195 PMC3232214

[cobi13835-bib-0049] Mounce, R. (2021). List of overlay journals. Version 0.1. Data set. Zenodo 10.5281/zenodo.5088734

[cobi13835-bib-0050] Munafò, M. R. , Nosek, B. A. , Bishop, D. V. M. , Button, K. S. , Chambers, C. D. , Percie Du Sert, N. , Simonsohn, U. , Wagenmakers, E. ‐. J. , Ware, J. J. , & Ioannidis, J. P. A. (2017). A manifesto for reproducible science. Nature Human Behaviour, 1, 0021. 10.1038/s41562-016-0021 PMC7610724 33954258

[cobi13835-bib-0051] Myers, N. , Mittermeier, R. A. , Mittermeier, C. G. , Da Fonseca, G. A. B. , & Kent, J. (2000). Biodiversity hotspots for conservation priorities. Nature, 403, 853–858. 10706275 10.1038/35002501

[cobi13835-bib-0052] Nguyen, V. M. , Brooks, J. L. , Young, N. , Lennox, R. J. , Haddaway, N. , Whoriskey, F. G. , Harcourt, R. , & Cooke, S. J. (2017). To share or not to share in the emerging era of big data: Perspectives from fish telemetry researchers on data sharing. Canadian Journal of Fisheries and Aquatic Sciences, 74, 1260–1274.

[cobi13835-bib-0053] Nosek, B. A. , Alter, G. , Banks, G. C. , Borsboom, D. , Bowman, S. D. , Breckler, S. J. , Buck, S. , Chambers, C. D. , Chin, G. , Christensen, G. , Contestabile, M. , Dafoe, A. , Eich, E. , Freese, J. , Glennerster, R. , Goroff, D. , Green, D. P. , Hesse, B. , Humphreys, M. , & Ishiyama, J. (2015). Promoting an open research culture. Science, 348, 1422–1425. 26113702 10.1126/science.aab2374 PMC4550299

[cobi13835-bib-0054] Nosek, B. A. , & Lakens, D. (2014). Registered reports: A method to increase the credibility of published results. Social Psychology, 45, 137–141.

[cobi13835-bib-0055] O'dea, R. E. , Parker, T. H. , Chee, Y. En , Culina, A. , Drobniak, S. M. , Duncan, D. H. , Fidler, F. , Gould, E. , Ihle, M. , Kelly, C. D. , Lagisz, M. , Roche, D. G. , Sánchez‐Tójar, A. , Wilkinson, D. P. , Wintle, B. C. , & Nakagawa, S. (2021). Towards open, reliable, and transparent ecology and evolutionary biology. BMC Biology, 19, 68. 33836762 10.1186/s12915-021-01006-3 PMC8034279

[cobi13835-bib-0056] Olijhoek, T. , & Tennant, J. (2018). The “problem” of predatory publishing remains a relatively small one and should not be allowed to defame open access. Available from https://blogs.lse.ac.uk/impactofsocialsciences/2018/09/25/the-problem-of-predatory-publishing-remains-a-relatively-small-one-and-should-not-be-allowed-to-defame-open-access/

[cobi13835-bib-0058] Parker, T. , Fraser, H. , & Nakagawa, S. (2019). Making conservation science more reliable with preregistration and registered reports. Conservation Biology, 33, 747–750. 31074110 10.1111/cobi.13342

[cobi13835-bib-0059] Pérignon, C. , Gadouche, K. , Hurlin, C. , Silberman, R. , & Debonnel, E. (2019). Certify reproducibility with confidential data. Science, 365, 127–128. 31296759 10.1126/science.aaw2825

[cobi13835-bib-0060] Perkel, J. M. (2019). Make code accessible with these cloud services. Nature, 575, 247–248. 31690867 10.1038/d41586-019-03366-x

[cobi13835-bib-0061] Perkel, J. M. (2018). A toolkit for data transparency takes shape. Nature, 560, 513–515. 30127481 10.1038/d41586-018-05990-5

[cobi13835-bib-0062] Peterson, A. T , Anderson, R. P. , Beger, M. , Bolliger, J. , Brotons, L. , Burridge, C. P. , Cobos, M. E. , Cuervo‐Robayo, A. P. , Di Minin, E. , Diez, J. , Elith, J. , Embling, C. B. , Escobar, L. E. , Essl, F. , Feeley, K. J. , Hawkes, L. , Jiménez‐García, D. , Jimenez, L. , Green, D. M. , … Kühn, I. (2019). Open access solutions for biodiversity journals: Do not replace one problem with another. Diversity and Distributions, 25, 5–8.

[cobi13835-bib-0063] Piwowar, H. , Priem, J. , Larivière, V. , Alperin, J. P. , Matthias, L. , Norlander, B. , Farley, A. , West, J. , & Haustein, S. (2018). The state of OA: A large‐scale analysis of the prevalence and impact of open access articles. PeerJ, 6, e4375. 29456894 10.7717/peerj.4375 PMC5815332

[cobi13835-bib-0064] Poisot, T. , Bruneau, A. , Gonzalez, A. , Gravel, D. , & Peres‐Neto, P. (2019). Ecological data should not be so hard to find and reuse. Trends in Ecology & Evolution, 34, 494–496. 31056219 10.1016/j.tree.2019.04.005

[cobi13835-bib-0065] Poisot, T. , Mounce, R. , & Gravel, D. (2013). Moving toward a sustainable ecological science: Don't let data go to waste! Ideas in Ecology and Evolution, 6, 11–19.

[cobi13835-bib-0066] Reid, A. J. , Eckert, L. E. , Lane, J.‐F. , Young, N. , Hinch, S. G. , Darimont, C. T. , Cooke, S. J. , Ban, N. C. , & Marshall, A. (2021). “Two‐Eyed Seeing”: An Indigenous framework to transform fisheries research and management. Fish and Fisheries, 22, 243–261.

[cobi13835-bib-0067] Rey, S. J. (2014). Open regional science. Annals of Regional Science, 52, 825–837.

[cobi13835-bib-0068] Robinson, O. J. , Ruiz‐Gutierrez, V. , Reynolds, M. D. , Golet, G. H. , Strimas‐Mackey, M. , & Fink, D. (2020). Integrating citizen science data with expert surveys increases accuracy and spatial extent of species distribution models. Diversity and Distributions, 26, 976–986.

[cobi13835-bib-0069] Roche, D. G. , Bennett, J. R. , Provencher, J. , Rytwinski, T. , Haddaway, N. R. , & Cooke, S. J. (2019). Environmental sciences benefit from robust evidence irrespective of speed. Science of the Total Environment, 696, 134000. 31465915 10.1016/j.scitotenv.2019.134000

[cobi13835-bib-0070] Roche, D. G. , Granados, M. , Austin, C. C. , Wilson, S. , Mitchell, G. M. , Smith, P. A. , Cooke, S. J. , & Bennett, J. R. (2020). Open government data and environmental science: A federal Canadian perspective. FACETS, 5, 942–962.

[cobi13835-bib-0071] Roche, D. G. , Lanfear, R. , Binning, S. A. , Haff, T. M. , Schwanz, L. E. , Cain, K. E. , Kokko, H. , Jennions, M. D. , & Kruuk, L. E. B. (2014). Troubleshooting public data archiving: Suggestions to increase participation. PLoS Biology, 12, e1001779. 24492920 10.1371/journal.pbio.1001779 PMC3904821

[cobi13835-bib-0072] Sarewitz, D. (2015). CRISPR: Science can't solve it. Nature, 522, 413–414. 26108836 10.1038/522413a

[cobi13835-bib-0073] Science Europe . (2021). The OA diamond journals study. Part 2: Recommendations. Available from 10.5281/zenodo.4562790

[cobi13835-bib-0074] Siler, K. (2020). Demarcating spectrums of predatory publishing: Economic and institutional sources of academic legitimacy. Journal of the Association for Information Science and Technology, 71, 1386–1401.

[cobi13835-bib-0075] Soeharjono, S. , & Roche, D. G. (2021). Reported individual costs and benefits of sharing open data among Canadian faculty members in ecology and evolution. BioScience, 71, 750–756.

[cobi13835-bib-0076] Soulé, M. E. (1985). What is conservation biology? Bioscience, 35, 727–734.

[cobi13835-bib-0077] Stodden, V. (2010). Open science: Policy implications for the evolving phenomenon of user‐led scientific innovation. Journal of Science Communication, 09, A05.

[cobi13835-bib-0078] Stodden, V. , Mcnutt, M. , Bailey, D. H. , Deelman, E. , Gil, Y. , Hanson, B. , Heroux, M. A. , Ioannidis, J. P. A. , & Taufer, M. (2016). Enhancing reproducibility for computational methods. Science, 354, 1240–1241. 27940837 10.1126/science.aah6168

[cobi13835-bib-0079] Sullivan, B. L. , Phillips, T. , Dayer, A. A. , Wood, C. L. , Farnsworth, A. , Iliff, M. J. , Davies, I. J. , Wiggins, A. , Fink, D. , Hochachka, W. M. , Rodewald, A. D. , Rosenberg, K. V. , Bonney, R. , & Kelling, S. (2017). Using open access observational data for conservation action: A case study for birds. Biological Conservation, 208, 5–14.

[cobi13835-bib-0080] Tai, T. C. , & Robinson, J. P. W. (2018). Enhancing climate change research with open science. Frontiers in Environmental Science, 6, 115.

[cobi13835-bib-0081] Tennant, J. P. , Waldner, F. , Jacques, D. C. , Masuzzo, P. , Collister, L B. , & Hartgerink, C. H. J. (2016). The academic, economic and societal impacts of open access: An evidence‐based review. F1000Research, 5, 632–632. 27158456 10.12688/f1000research.8460.1 PMC4837983

[cobi13835-bib-0082] Tenopir, C. , Allard, S. , Douglass, K. , Aydinoglu, A. U. , Wu, L. , Read, E. , Manoff, M. , & Frame, M. (2011). Data sharing by scientists: Practices and perceptions. PLoS One, 6, e21101. 21738610 10.1371/journal.pone.0021101 PMC3126798

[cobi13835-bib-0083] Tenopir, C. , Dalton, E. D. , Allard, S. , Frame, M. , Pjesivac, I. , Birch, B. , Pollock, D. , & Dorsett, K. (2015). Changes in data sharing and data reuse practices and perceptions among scientists worldwide. PLoS One, 10, e0134826. 26308551 10.1371/journal.pone.0134826 PMC4550246

[cobi13835-bib-0084] Turner, W. , Rondinini, C. , Pettorelli, N. , Mora, B. , Leidner, A. K. , Szantoi, Z. , Buchanan, G. , Dech, S. , Dwyer, J. , Herold, M. , Koh, L. P. , Leimgruber, P. , Taubenboeck, H. , Wegmann, M. , Wikelski, M. , & Woodcock, C. (2015). Free and open‐access satellite data are key to biodiversity conservation. Biological Conservation, 182, 173–176.

[cobi13835-bib-0201] UNESCO (2019). Recommendation on Open Educational Resources (OER). Available from http://portal.unesco.org/en/ev.php‐URL_ID=49556&URL_DO=DO_TOPIC&URL_SECTION=201.html

[cobi13835-bib-0085] Walsh, J. C. , Dicks, L. V. , Raymond, C. M. , & Sutherland, W. J. (2019). A typology of barriers and enablers of scientific evidence use in conservation practice. Journal of Environmental Management, 250, 109481. 31518795 10.1016/j.jenvman.2019.109481

[cobi13835-bib-0086] Ware, M. , & Mabe, M. (2015). The STM report: An overview of scientific and scholarly journal publishing. DigitalCommons@University of Nebraska ‐ Lincoln. Available from. https://digitalcommons.unl.edu/scholcom/9/?utm_source=digitalcommons.unl.edu%2Fscholcom%2F9&utm_medium=PDF&utm_campaign=PDFCoverPages

[cobi13835-bib-0087] White, E. , Baldridge, E. , Brym, Z. , Locey, K. , Mcglinn, D. , & Supp, S. (2013). Nine simple ways to make it easier to (re) use your data. Ideas in Ecology and Evolution, 6, 1–10.

[cobi13835-bib-0088] Whitehead, A. L. , & Booker, D. J. (2019). Communicating biophysical conditions across New Zealand's rivers using an interactive webtool. New Zealand Journal of Marine and Freshwater Research, 53, 278–287.

[cobi13835-bib-0089] Wilkinson, M. D. , Dumontier, M. , Aalbersberg, I. J. , Appleton, G. , Axton, M. , Baak, A. , Blomberg, N. , Boiten, J. ‐. W. , Da Silva Santos, L. B. , Bourne, P E. , Bouwman, J. , Brookes, A. J. , Clark, T. , Crosas, M. , Dillo, I. , Dumon, O. , Edmunds, S. , Evelo, C. T. , Finkers, R. , & Gonzalez‐Beltran, A. (2016). The FAIR Guiding Principles for scientific data management and stewardship. Scientific Data, 3, 160018. 26978244 10.1038/sdata.2016.18 PMC4792175

[cobi13835-bib-0090] Willinsky, J. , & Rusk, M. (2019). If research libraries and funders finance open access: Moving beyond subscriptions and APCs. College & Research Libraries, 80, 340.

[cobi13835-bib-0091] Wong, C. , Ballegooyen, K. , Ignace, L. , Johnson, M. J. G. , & Swanson, H. (2020). Towards reconciliation: 10 Calls to Action to natural scientists working in Canada. FACETS, 5, 769–783.

[cobi13835-bib-0092] Young, N. , Corriveau, M. , Nguyen, V. M. , Cooke, S. J. , & Hinch, S. G. (2016). How do potential knowledge users evaluate new claims about a contested resource? Problems of power and politics in knowledge exchange and mobilization. Journal of Environmental Management, 184, 380–388. 27745770 10.1016/j.jenvman.2016.10.006

[cobi13835-bib-0093] Yuan, L. , MacNeill, S. , & Kraan, W. G. (2008). Open educational resources—Opportunities and challenges for higher education. Educational Cybernetics: Reports, Paper 1. Available from: http://digitalcommons.bolton.ac.uk/iec_reports/1

