# Supplementary material for: Closing the knowledge‐action gap in conservation with open science
Source: Conserv Biol. 2021 Nov 29;36(3):e13835. doi: 10.1111/cobi.13835 (PMC9300006; doi:10.1111/cobi.13835)
Supplement: Supplementary file 1 — Supporting Information Additional information is available online in the Supporting Information section at the end of the online article. The authors are solely responsible for the content and functionality of these materials. Queries (other than absence of the material) should be directed to the corresponding author. Appendix S1. Example of open data for conservation science and practice: the TEAM Network Appendix S2. Open resources to help with evidence synthesis in conservation science and practice Appendix S3. Example of open software for conservation science and practice: Marxan Connect [file COBI-36-0-s001.pdf]

SUPPORTING INFORMATION

Roche, D. G., O'Dea, R. E., Kerr, K. A., Rytwinski, T., Schuster, R., Nguyen, V. M., Young, N., Bennett, J. R., & Cooke, S. J. (2021). Closing the knowledge-action gap in conservation with open science. *Conservation Biology*. https://doi.org/10.1111/cobi.13835

Appendix S1. Example of open data for conservation science and practice: the TEAM Network

Established in 2002, the Tropical Ecology, Assessment, and Monitoring (TEAM) Network is a partnership among Conservation International, The Wildlife Conservation Society and the Smithsonian Institution designed to provide an early warning system for biodiversity loss in tropical forests (Fonseca & Benson 2003). Using standardized monitoring protocols for climate, terrestrial vertebrates, and vegetation, TEAM collects near-real time data for monitoring long-term trends in the status of biodiversity, climate, and human disturbance through a global network of field stations (TEAM Sites) located in Africa, Asia, and Latin America (Rovero & Ahumada 2017). The TEAM network is the most extensive tropical camera-trap program in the world, systematically monitoring >500 populations of ground dwelling mammals and birds in 17 tropical forests (Beaudrot et al. 2019). Data and code are open and used to evaluate wildlife trends within and across protected areas and to assess the effectiveness of protected areas in maintaining wildlife populations. For example, Ahumada et al. (2013) reported that three of 13 mammal species monitored at a TEAM site in Costa Rica showed significant declines in occupancy over a 5-year period, hypothesizing that two of these species were declining due to illegal hunting. After sharing these study results with park authorities, measures were taken to increase patrolling and control poaching in the protected area. Monitoring data collected following the enforcement of the conservation actions showed that these two species had since stabilized (Ahumada et al. 2016). The TEAM's approach of sharing data and platforms that provide summary analyses such as the Wildlife Picture Index (WPI; a robust biodiversity indicator developed for camera-trap data and designed to evaluate composite biodiversity trends), have the potential to greatly increase the range of TEAM data users and uses (Rovero & Ahumada 2017). Previously, the TEAM network data and analytics systems were accessible via the TEAM network website (www.teamnetwork.org); they are now being migrated to Wildlife Insights (www.wildlifeinsights.org/team-network), with an expected release in summer 2021.

Other examples of open data for conservation science and practice include GBIF (www.gbif.org), iNaturalist (www.inaturalist.org), eBird (https://ebird.org), GEO BON (https://geobon.org), IUCN Red List (https://www.iucnredlist.org), North American Breeding Bird Survey (www.pwrc.usgs.gov/bbs).

Appendix S2. Open resources to help with evidence synthesis in conservation science and practice

Conservation Evidence (www.conservationevidence.com) is an open resource designed to support those making conservation policy and management decisions. The website provides evidence summaries from the scientific literature (>7000 study summaries available) on the effectiveness of conservation actions for maintaining and restoring biodiversity (>2500 species or habitat management actions) (Sutherland & Wordley 2018). Conservation Evidence also produces synopses of evidence that review the effectiveness of all actions a decision maker could implement to conserve a given species group, habitat, or to tackle a particular conservation issue, with 20 subject-wide evidence syntheses available to-date (e.g., bee conservation, forest conservation, sustainable aquaculture). Similarly, the Collaboration for Environmental Evidence Database of Evidence Reviews (CEEDER; www.environmentalevidence.org/ceeder) is an open evidence resource that helps evidence consumers find reliable reviews and syntheses to inform their decision-making. With this open database, decision makers can search syntheses (evidence reviews and overviews) on a specific question of environmental policy or management relevance. Each synthesis is presented alongside an independent assessment of its reliability with respect to its use in decision-making (Konno et al. 2020). Open access evidence resources such as these support evidence translation and knowledge mobilisation to help close the knowledge-action gap.

Appendix S3. Example of open software for conservation science and practice: Marxan Connect

Marxan Connect is an open source software tool that provides a Graphical User Interface (GUI) for Marxan (Daigle et al. 2020), one of the most-widely used spatial planning tools delivering outcomes for conservation planning in over 100 countries worldwide (Sinclair et al. 2018). Marxan helps practitioners find potential optimum solutions for protected areas networks, include connectivity in their network planning, evaluate the performance of protected area networks, and develop multiple-use zoning plans. One challenge of using the software is it requires technical skills to run systematic conservation planning analyses. Marxan Connect was developed to close this capacity gap by allowing scientists and practitioners without coding skills to use Marxan. Both Marxan and Marxan Connect are open source and available on GitHub (https://github.com/Marxan-source-code, https://github.com/remi-daigle/MarxanConnect), allowing users to understand the inner workings of the software and contribute to both projects if they wish to do so. Marxan Connect users can save project files directly from the GUI (JSON formatted text that stores all filepaths, options, and connectivity metrics defined by the user), allowing them to save and share their analysis with other users. Marxan Connect also provides a python module called ‘marxanconpy’, which enables command line interaction with Marxan Connect functions. This command line interface encourages transparent and reproducible workflows by allowing users to save and share their analysis code.

Other notable examples of open software for conservation science and practice include prioritizr (https://prioritizr.net), Maxent (https://biodiversityinformatics.amnh.org), Circuitscape (https://circuitscape.org), Wild Me (https://wildme.org), First Observer (https://github.com/persts/FirstObserver), BIOMOD (http://www.will.chez-alice.fr/Software.html), and Zonation (https://conservationcorridor.org).

References

Ahumada JA, Hurtado J, Lizcano D. 2013. Monitoring the status and trends of tropical forest terrestrial vertebrate communities from camera trap data: a tool for conservation. PloS One **8**:e73707.

Ahumada JA, O'brien TG, Mugerwa B, Hurtado J, Rovero F, Zimmermann F. 2016. Camera trapping as a monitoring tool at national and global levels. Pages 196–218 in Rovero F, and Zimmermann F, editors. Camera Trapping for Wildlife Research. Pelagic Publishing Ltd, Exeter, UK.

Beaudrot L, Ahumada J, O'Brien TG, Jansen PA. 2019. Detecting tropical wildlife declines through camera-trap monitoring: an evaluation of the Tropical Ecology Assessment and Monitoring protocol. Oryx **53**:126-129.

Daigle RM, Metaxas A, Balbar AC, McGowan J, Treml EA, Kuempel CD, Possingham HP, Beger M. 2020. Operationalizing ecological connectivity in spatial conservation planning with Marxan Connect. Methods in Ecology and Evolution **11**:570-579.

Fonseca G, Benson PJ. 2003. Biodiversity conservation demands open access. PLOS Biology **1**:e46.

Konno K, Cheng SH, Eales J, Frampton G, Kohl C, Livoreil B, Macura B, O'Leary BC, Randall NP, Taylor JJ. 2020. The CEEDER database of evidence reviews: An open-access evidence service for researchers and decision-makers. Environmental Science & Policy **114**:256-262.

Rovero F, Ahumada J. 2017. The Tropical Ecology, Assessment and Monitoring (TEAM) Network: An early warning system for tropical rain forests. Science of The Total Environment **574**:914-923.

Sinclair SP, Milner-Gulland E, Smith RJ, McIntosh EJ, Possingham HP, Vercammen A, Knight AT. 2018. The use, and usefulness, of spatial conservation prioritizations. Conservation Letters **11**:e12459.

Sutherland WJ, Wordley CFR. 2018. A fresh approach to evidence synthesis. Nature **558**:364–366.
